# Glucagon-Like Peptide-1 Secreting Cell Function as well as Production of Inflammatory Reactive Oxygen Species Is Differently Regulated by Glycated Serum and High Levels of Glucose

**DOI:** 10.1155/2014/923120

**Published:** 2014-02-04

**Authors:** Alessandra Puddu, Roberta Sanguineti, Fabrizio Montecucco, Giorgio L. Viviani

**Affiliations:** ^1^Department of Internal Medicine, University of Genoa School of Medicine, IRCCS Azienda Ospedaliera Universitaria San Martino, IST Istituto Nazionale per la Ricerca sul Cancro, 6 Viale Benedetto XV, 16143 Genoa, Italy; ^2^Division of Cardiology, Department of Medicine, Geneva University Hospitals, Faculty of Medicine, Foundation for Medical Researches, 64 Avenue de la Roseraie, 1211 Geneva, Switzerland; ^3^Division of Laboratory Medicine, Department of Genetics and Laboratory Medicine, Geneva University Hospitals, 4 Rue Gabrielle-Perret-Gentil, 1205 Geneva, Switzerland

## Abstract

Glucagon-like peptide-1 (GLP-1), an intestinal hormone contributing to glucose homeostasis, is synthesized by proglucagon and secreted from intestinal neuroendocrine cells in response to nutrients. GLP-1 secretion is impaired in type 2 diabetes patients. Here, we aimed at investigating whether diabetic toxic products (glycated serum (GS) or high levels of glucose (HG)) may affect viability, function, and insulin sensitivity of the GLP-1 secreting cell line GLUTag. Cells were cultured for 5 days in presence or absence of different dilutions of GS or HG. GS and HG (alone or in combination) increased reactive oxygen species (ROS) production and upregulated proglucagon mRNA expression as compared to control medium. Only HG increased total production and release of active GLP-1, while GS alone abrogated secretion of active GLP-1. HG-mediated effects were associated with the increased cell content of the prohormone convertase 1/3 (PC 1/3), while GS alone downregulated this enzyme. HG upregulated Glucokinase (GK) and downregulated SYNTHAXIN-1. GS abrogated SYNTHAXIN-1 and SNAP-25. Finally, high doses of GS alone or in combination with HG reduced insulin-mediated IRS-1 phosphorylation. In conclusion, we showed that GS and HG might regulate different pathways of GLP-1 production in diabetes, directly altering the function of neuroendocrine cells secreting this hormone.

## 1. Introduction

Glucagon-like peptide-1 (GLP-1) is an intestinal hormone regulating glycaemia via the increase of insulin and concomitant reduction of glucagon secretion, the salvage of beta-cell from apoptosis, the regulation of gastric emptying, and food intake [1–3]. GLP-1 is synthesized by posttranslational processing of proglucagon and is secreted from specialized intestinal neuroendocrine cells, the L-cells, in response to dietary nutrients (particularly carbohydrates and lipids) [4, 5]. After the demonstration of GLP-1 as a promising molecule in the treatment of T2DM, there was an intense debate on the pathophysiological relevance of GLP-1 in diabetes [6–10]. Rask and coworkers demonstrated in a cohort of asymptomatic subjects that insulin resistance is negatively correlated with GLP-1 secretion [11, 12]. Consistent with these findings, Lim and colleagues demonstrated that GLP-1 is secreted in response to insulin, suggesting that insulin resistance might be associated with alteration in GLP-1 exocytosis [13]. Several studies revealed that a long-lasting deleterious effect of hyperglycemia persists when glycemic control has been achieved and defined this phenomenon as the “metabolic memory” [14–16]. It is well known that hyperglycemia enhances the endogenous nonenzymatic glycosylation of proteins, lipids, and nucleic acids, a process that may result in the accumulation of heterogeneous molecules known as advanced glycation end products (AGEs) that are increased in the glycated serum (GS) [17, 18]. Several studies showed a strict correlation between the accelerated formation of AGEs and the complications of diabetes [19–22] and proposed that the “metabolic memory” might be explained by persistent overproduction of reactive oxygen species (ROS) directly induced by AGEs [23–25]. Given the importance of GLP-1-mediated beneficial activities to restore normal glucose homeostasis in diabetes, we aimed at investigating whether GS and high glucose (HG) levels might affect *in vitro* viability, function, and insulin resistance in the GLP-1 secreting GLUTag cell line [26].

## 2. **Materials and Methods**

### 2.1. GS Preparation

GS was prepared by adding 50 mmol/L ribose to heat-inactivated (56°C for one hour) FBS, as described previously [27]. Aliquots of FBS were processed the same way but without ribose (nonglycated serum, NGS) and used for standard medium preparation. Pentosidine content was evaluated as a measure of protein glycation, as previously described [28]. The concentration of pentosidine in the experimental media ranged between 400 (GS) and 800 nmol/L (2GS), which corresponds to the levels within the pathophysiological range detected in the plasma of diabetic patients [29].

### 2.2. Cell Culture and Stimulation

GLUTag cells (kindly provided by Dr. FM. Gribble, Cambridge Institute for Medical Research, Department of Clinical Biochemistry, Cambridge, UK, with permission of Dr. D. Drucker, University of Toronto, ON, Canada) secrete GLP-1 in response to a number of neurotransmitters and nutrients [26]. For proliferation maintenance, cells were grown in DMEM (5.6 mmol/L glucose) supplemented with 10% fetal bovine serum (FBS), 2 mmol/L L-glutamine, 100 IU/mL penicillin, and 100 µg/mL streptomycin. Before each experiment the cells were split into 6-well plates and cultured for 5 days at different culture conditions: DMEM low glucose (5.6 mmol/L) (CTR) or DMEM high glucose (25 mmol/L) (HG) supplemented with different concentrations of GS.

### 2.3. Reactive Oxygen Species Detection

Intracellular reactive oxygen species (ROS) level was measured using the cell-permeable fluorescent probe, 2′,7′-dichlorofluorescein diacetate (DCFH-DA) (Sigma-Aldrich, Milan, Italy). In brief, cells were seeded into 6-well culture plates at 2 × 10^5^ cells/well and treated for 5 days as previously described, then washed twice with Hank's Buffered Salt Solution (HBSS), and incubated with fresh DCFH-DA (25 *μ*M) in HBSS for 30 min at 37°C in 5% CO_2_. After that, cells were washed twice in HBSS, and wells were filled with 1 mL HBSS before fluorescence acquisition in a plate reader (TECAN InfinitePro200) (Ex: *λ*485/Em: *λ*535 nm). Fluorescent emission was normalized to total protein content. Results were expressed as percentage of fluorescence compared to control (CTR) (100%).

### 2.4. Cell Viability Assay

Cells were seeded into 96-well culture plates at 2 × 10^4^ cells/well and treated for 5 days. Cell proliferation rate was determined using the Cell Titer 96 Aqueous One Solution Cell Proliferation Assay (PROMEGA ITALIA, Milan, Italy) according to the manufacturer's instructions. Briefly, this is a colorimetric method determining the number of viable cells via MTS tetrazolium reduction measured through formazan production. The MTS tetrazolium compound is bioreduced by cells into a colored formazan product directly proportional to the number of living cells in culture. Values were expressed as the percentage of absorbance compared to CTR (100%).

### 2.5. Reverse Transcriptase Polymerase Chain Reaction

Total RNA was extracted from GLUTag cells with RNeasy kit (QIAGEN s.r.l., Milan, Italy) according to manufacturer's instruction. The RNA concentrations were determined spectrophotometrically and equal quantities of total RNA were used from different samples. One microgram of RNA was reverse-transcripted to cDNA using GoScript Reverse Transcription System (PROMEGA ITALIA, Milan, Italy). Then PCR amplification was performed using the following specific primers: proglucagon sense TGAAGACCATTTACTTTGTGGCT, antisense TGGTGGCAAGATTATCCAGAAT; GAPDH sense TCCACCCTGTTGCTGTAG, antisense GACCACAGTCCATGCCATCACT. All the samples were amplified in a linear amplification range established using a serial cDNA dilution and varying the number of cycles (28 cycles for GAPDH, 30 for proglucagon). The predicted band sizes (bp) of the fragments were as follows: proglucagon, 493; GAPDH, 453. PCR products were electrophoresed onto a 1.5% agarose gel containing EuroSafe Nucleic Acid Stain (EUROCLONE S.p.A, Milan, Italy) and visualized under UV light. The relative intensities of the bands were quantified by densitometric analysis. Results were shown as percentage change from CTR level (100%).

### 2.6. Measurements of GLP-1 Production and Secretion

Cells were lysed using RIPA buffer (Sigma-Aldrich, Milan, Italy) and lysates analysed for GLP-1 content. GLP-1 was assayed in supernatants and lysates using an active GLP-1 ELISA-kit (Millipore Corporation, Billerica, MA, USA). The means of assay variation of the ELISA kits were intra-assay CV 8% and interassay CV 9%. Total culture GLP-1 production was calculated as the sum of the media and cell content of GLP-1.

To study GLP-1 secretion, cells were washed with Hanks' balanced salt solution and incubated for 2 hours with medium containing 0.5% FBS. After the 2 h treatment, media were collected and DPP-4 inhibitor was added (10 µL/mL) (Millipore Corporation, Billerica, MA, USA). Then media were centrifuged to remove contaminating cells and stored at −80°C until the ELISA assay was performed. Secretion was normalized to protein content of the corresponding cell lysate. Secretion was calculated as the ratio between amount of GLP-1 in the medium and total culture content of GLP-1. Results for both total and released GLP-1 were shown as percentage change from CTR level (100%).

### 2.7. Immunoblot

At the end of the experiments GLUTag cells were lysed in RIPA buffer (50 mM Tris HCl pH 7.5, 150 mM NaCl, 1% NP40, 0.1% SDS, supplemented with protease and phosphatase inhibitors), and protein concentrations were determined using the BCA Protein Assay Kit. Thirty micrograms of total cell lysate was separated on a SDS-PAGE and transferred onto nitrocellulose. Filters were blocked in 5% BSA and incubated overnight at 4°C with primary specific antibodies (anti-SNAP-25, anti-Syntaxin-1, anti-Glucokinase, and anti-*β*-Actin from Santa Cruz Biotechnology, Inc. Santa Cruz, CA, USA; anti-PC 1/3 from Millipore, Billerica, MA, USA; anti-Insulin Receptor Substrate-1 [IRS-1], Phospho-IRS-1 Tyr895, and Insulin Receptor *β* [IR] from Cell Signaling Technology, Beverly, MA, USA). Secondary specific horseradish-peroxidase linked antibodies were added for 1 hour at room temperature. Bound antibodies were detected using an enhanced chemiluminescence lighting system (Luminata Classico, Millipore, Billerica, MA, USA), according to manufacturer's instruction. To verify equal loading of the proteins, membranes were stripped, reblocked, and reprobed to detect *β*-actin. Values of proteins of interest were normalized to total amounts of *β*-actin. To evaluate specific phosphorylation of IRS-1 at Tyr895 GLUTag cells were cultured 5 days with AGEs or high glucose concentration, washed, incubated for 2 hours in serum-free medium, and finally exposed for 5 min to 100 nmol/L insulin. Cells were then lysed and lysates separated by SDS-PAGE and immunoblotted with specific antibody anti-phospho IRS-1 (Tyr895). Membranes were stripped and reprobed with anti-IRS-1 antibody to normalize the blots for the total protein levels. Bands of interest were quantified by densitometry using the NIH program ImageJ. Results were expressed as percentages of CTR (defined as 100%).

### 2.8. Statistical Analysis

The results were representative of at least 3 experiments. All analyses were carried out with the GraphPad Prism 4.0 software (GraphPad Software, San Diego, CA, USA). Data were expressed as the mean ± SE. Comparison between control and single treatments was done using Dunnett's test. *P* value < 0.05 was considered as statistically significant.

## 3. Results

### 3.1. GS and HG Increase ROS Intracellular Production without Affecting Cell Viability

**Figure 1:**
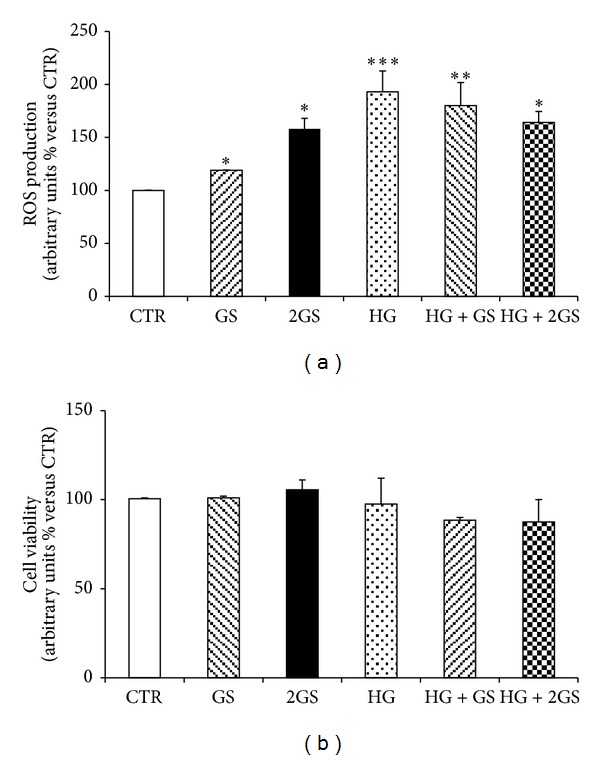
GS and HG increase intracellular ROS without affecting GLUTag cell viability. After 5-day treatment in the presence of standard medium (CTR) or high glucose concentration (25 mmol/L) (HG) in presence of different concentrations of glycated serum (400 [GS] and 800 nmol/L [2GS]), ROS intracellular production (a) and cell viability (b) were assessed. Values shown indicate the percentage of absorbance compared to CTR. Data are expressed as the mean ± SE of *n* = 5 (ROS release) or *n* = 3 (cell viability) independent experiments. *P < 0.05, **P < 0.01, and ***P < 0.001 versus CTR.

ROS have been shown to potentially mediate lipoapoptosis of GLP-1-secreting cells [30] and increase AGE-mediated damages in other cell systems [31, 32]. Incubation with different concentrations of GS (within the pathophysiological range in plasma of diabetic patients) [29] or high glucose (HG) significantly increases ROS intracellular production as compared to cells incubated with control medium (Figure 1(a)). However, concomitant stimulation with GS and HG does not alter ROS production as compared to HG. No difference in cell viability was shown in GLUTag cells cultured under the same conditions (Figure 1(b)).

### 3.2. GS and HG Differently Regulate GLUTag Cell Function

**Figure 2:**
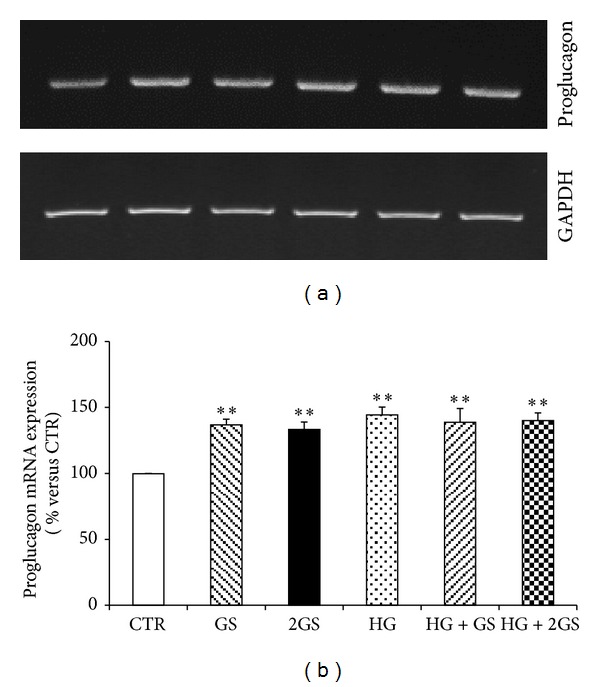
GS and HG increase proglucagon mRNA expression. Semiquantitative RT-PCR of proglucagon mRNA expression in GLUTag cells stimulated standard medium (CTR) or high glucose concentration (25 mmol/L) (HG) in presence of different concentrations of glycated serum (400 [GS] and 800 nmol/L [2GS]). (a) Representative agarose gel. (b) Quantification of densitometries of agarose gel bands. Data are represented as mean ± SE (*n* = 3). **P < 0.01 versus CTR.

**Figure 3:**
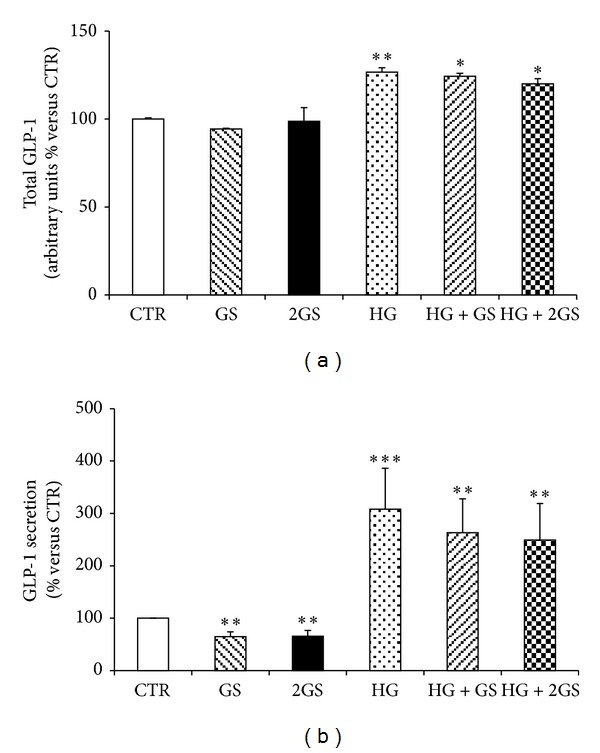
HG increases GLP-1 total production and release. Levels of total GLP-1 production (a) (calculated as the sum of the supernatant and cell content of GLP-1) and GLP-1 released (b) in the supernatants were shown. Data are expressed as mean ± SE (*n* = 5). *P < 0.05, **P < 0.01, and ***P < 0.001 versus CTR.

In order to investigate whether incubation with GS and HG affects *in vitro* GLUTag cell function, the cell expression of proglucagon and the production and release of GLP-1 were investigated. Proglucagon mRNA expression was significantly upregulated in presence of different doses of GS and HG (or combination of both) when compared to cells incubated with control medium (Figures 2(a) and 2(b)). In the same cells, only HG (alone or in the presence of GS) upregulated total GLP-1 production as compared to controls (Figure 3(a)). No effect was shown for incubation with GS (Figure 3(a)). Accordingly, incubation with HG levels (alone or in the presence of GS) significantly increased GLP-1 release in the cell supernatants (Figure 3(b)). Despite being statistically significant, a slight reduction in GLP-1 concentrations within GLUTag cell supernatants was shown in the presence of GS as compared to control medium (Figure 3(b)).

**Figure 4:**
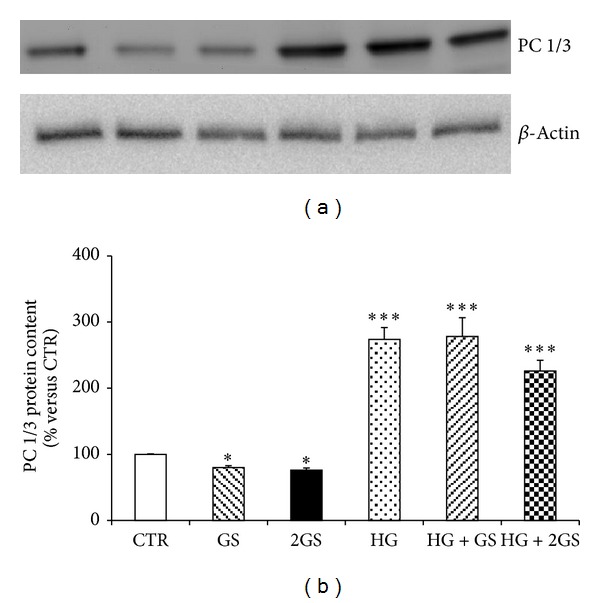
HG increases PC 1/3 protein levels, while GS decreases them. (a) Representative western blot analysis. (b) Quantification of densitometries of western blot bands. Data were expressed as mean ± SE of fold induction relative to *β*-actin (*n* = 3). *P < 0.05 and ***P < 0.001 versus CTR.

**Figure 5:**
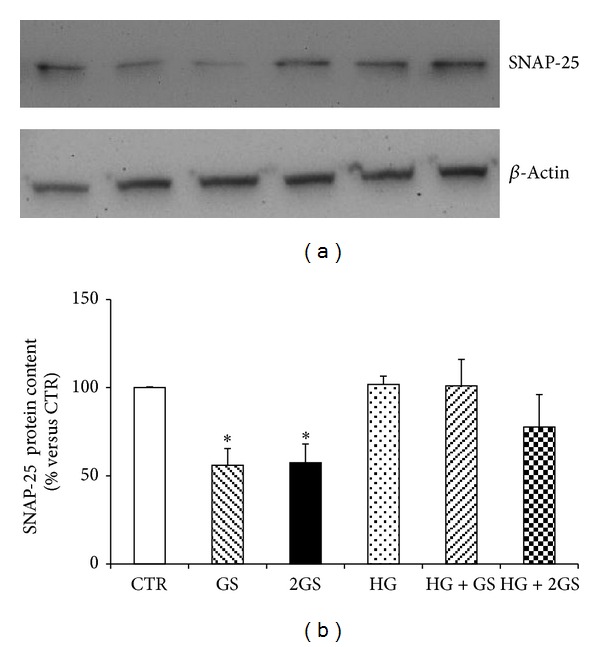
GS decreases SNAP-25 protein content. (a) Representative western blot analysis. (b) Quantification of densitometries of western blot bands. Data were expressed as mean ± SE of fold induction relative to *β*-actin (*n* = 4). *P < 0.05 versus CTR.

**Figure 6:**
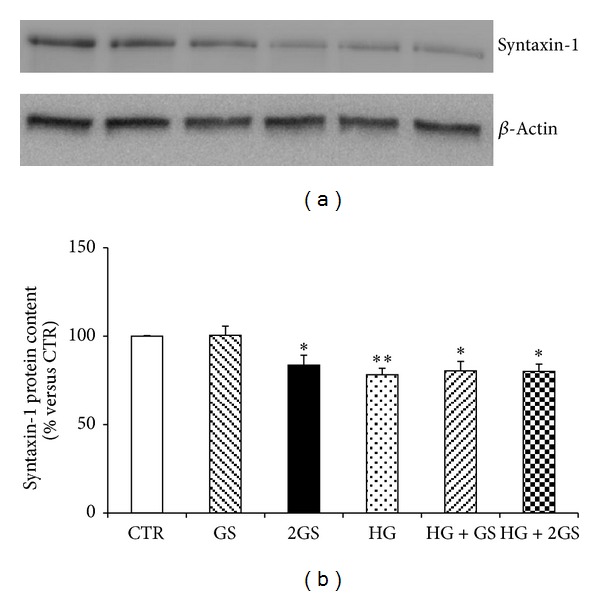
The higher concentrations of GS and HG decrease SYNTHAXIN-1 protein content. (a) Representative western blot analysis. (b) Quantification of densitometries of western blot bands. Data were expressed as mean ± SE of fold induction relative to *β*-actin (*n* = 6). *P < 0.05 and **P < 0.01 versus CTR.

**Figure 7:**
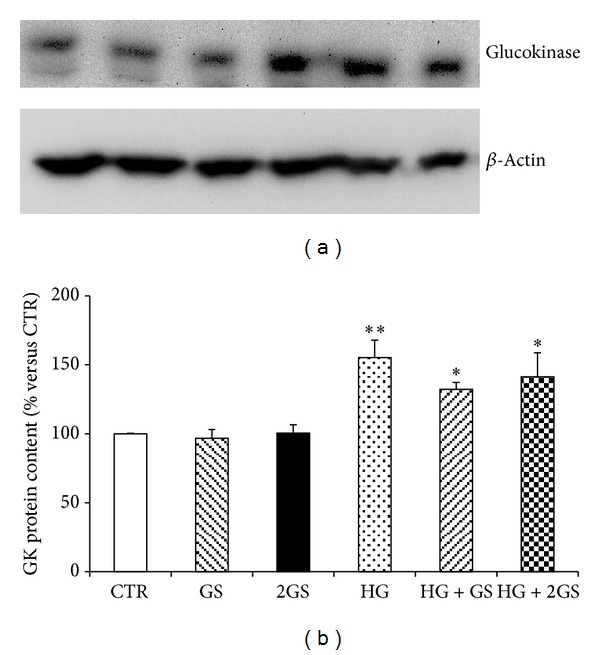
HG increases Glucokinase protein content. (a) Representative western blot analysis. (b) Quantification of densitometries of western blot bands. Data were expressed as mean ± SE of fold induction relative to *β*-actin (*n* = 4).*P < 0.05 and **P < 0.01 versus CTR.

In order to identify the molecular mechanisms triggered by the different stimuli in the regulation of GLUTag cell function, we focused on prohormone convertase 1/3 (PC1/3) that was shown to be a critical enzyme in the posttranslational processing of proglucagon to GLP-1 in L-cells [33, 34]. Incubation with GS was associated with a significant decrease in PC 1/3 protein expression levels in cells cultured at low glucose concentration (Figures 4(a) and 4(b)). On the other hand, PC 1/3 was significantly upregulated by HG incubation (alone or in the presence of GS) (Figures 4(a) and 4(b)). Since exocytosis of GLP-1 granules might be related to the docking and fusion activity of SNAP-25 and syntaxin-1 in GLUTag cells [35], we investigated the levels of these proteins in the cell cultures. SNAP-25 was significantly reduced by both concentrations of GS in cells cultured at low glucose concentration but was not affected by HG incubation (Figures 5(a) and 5(b)). Conversely, SYNTHAXIN-1 protein level was exclusively reduced by the highest concentration of GS in cells cultured at low glucose concentration and by HG (alone or in the presence of GS) (Figures 6(a) and 6(b)). In order to evaluate whether GLP-1 protein secretion was coupled to glucose metabolism, the protein levels of the glucose sensor Glucokinase (GK) were assessed in the same cell cultures. HG increased the GK levels as compared to control medium (Figures 7(a) and 7(b)). On the other hand, the expression of GK in cells cultured in HG was not affected by GS (Figures 7(a) and 7(b)).

### 3.3. GS and HG Differently Regulate the Response to Insulin in GLUTag Cells

**Figure 8:**
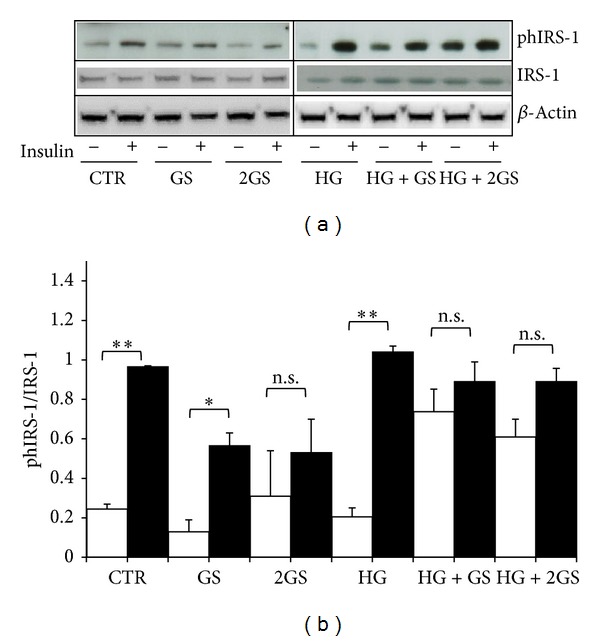
GS abrogates insulin-induced phosphorylation of IRS-1(Tyr-895) at both low and high glucose concentration. After 5-day treatment in the presence of standard medium (CTR) or high glucose concentration (25 mmol/L) (HG) in presence of different concentrations of glycated serum (400 [GS] and 800 nmol/L [2GS]), GLUTag cells were incubated for 2 hours in serum-free medium and, then, exposed in the presence (dark bars) or absence (white bars) of 100 nmol/L insulin for 5 min. (a) Representative western blot analysis. (b) Quantification of densitometries of western blot bands. Data were expressed as mean ± SE of fold induction relative to *β*-actin (*n* = 4). *P < 0.05 and **P < 0.01 versus absence of insulin; n.s.: nonsignificant.

Since AGEs were shown to induce insulin resistance in several cell types and insulin sensitivity has been correlated with GLP-1 secretion [9, 11, 36–39], we investigated whether glycated serum of high glucose might be associated with insulin resistance in GLUTag cells. As insulin-mediated function, we investigated the ability of the hormone to phosphorylate IRS-1 (Insulin Receptor Substrate-1), one of the main substrates of the Insulin Receptor (IR) in the insulin signalling cascade. Insulin-induced phosphorylation of IRS-1(Tyr895) in GLUTag cells was preserved when cells were incubated with control medium (as expected for a positive control), lower dose of glycated serum, and high glucose (Figures 8(a) and 8(b)). Conversely, insulin-induced phosphorylation of IRS-1(Tyr895) was not maintained in the presence of the highest dose of GS in cells cultured at low glucose concentration and in presence of both doses of GS under HG condition (Figures 8(a) and 8(b)). Similarly to control medium, cells cultured with HG alone display a significant increase of IRS-1 phosphorylation in response to insulin. To verify whether these results were due to an altered expression of IRS-1, its protein levels were evaluated in GLUTag cells. No significant change in IRS-1 levels was shown under the different culture conditions as compared to control medium.

## 4. Discussion

A main finding of this paper is that detrimental effects of GS are mainly evident when cells were cultured at low glucose concentration. Conversely, when stimulation with GS is under HG conditions, GS does not alter HG-mediated cell response except for insulin-induced IRS-1 phosphorylation.

Another important finding of this paper is represented by the demonstration of the increase in intracellular ROS production of GLUTag cells exposed to GS or HG without any modification on cell viability. Recently, Kappe and coworkers demonstrated that incubation with palmitate was able to increase lipotoxicity in GLUTag cells by increasing ROS production [30]. Surprisingly, we showed here that these events might be not related. However, our data showed a detrimental effect of both diabetic toxic products. In particular, GS-induced ROS production was also accompanied with a potential functional damage (decrease of GLP-1 secretion in GLUTag). Considering HG, ROS generation may be due to the glucose overload [40].

Despite the fundamental role of GLP-1 in maintaining glucose homeostasis, long-term regulation of GLP-1 secretion by neuroendocrine cells has been relatively neglected in both basic research and clinical studies in diabetes. In particular, it remained to investigate whether hyperglycaemia or the “metabolic memory” would directly affect the viability, function, and insulin sensitivity of these GLP-1 secreting cells. In this study, we provided evidence that diabetic toxic products (such as GS and HG) differently affected GLP-1 secreting cell function, resulting in apparently opposite effects on GLP-1 secretion.

In fact, despite the fact that both stimuli increased proglucagon mRNA expression, only HG leads to increased GLP-1 production. This discrepancy may be due to a major degradation of proglucagon mRNA or to a defective processing of proglucagon into GLP-1. The opposite effects of GS and HG on intracellular protein content of PC 1/3 (the enzyme responsible for the specific posttranslational processing of proglucagon into GLP-1 in intestinal L cells) [33, 34] might suggest that GLUTag cells might fail to maintain a sustained proglucagon conversion when exposed to GS.

Considering that the production of GLP-1 in cells exposed to GS was comparable to that observed in cells cultured in control medium alone, our results suggest that the decreased GLP-1 secretion might be due to a defect in its release. Similarly to other endocrine cells, intestinal L-cells store GLP-1 in intracellular granules that are released via exocytosis [41–43]. In 2009, Ohara-Imaizumi and coworkers reported that exocytosis of GLP-1 was closely similar to the insulin granule fusion from pancreatic beta-cells and that stimulation with glucose might evoke a biphasic granule exocytosis with the fusion of two types of granules [44]. This regulated exocytosis pathway of both neuronal and endocrine cells might depend on targeting of granules to specific membrane compartments and involve the mutual recognition of granule proteins with plasma membrane proteins. In particular, SYNTHAXIN-1 and SNAP-25 (resident in the plasma membrane) were recently shown to complex with synaptobrevin anchored on the granules. These proteins assemble into stable docking and fusion complexes also in GLUTag cells [35]. Altered expression and localization of SYNTHAXIN-1 and SNAP-25 have been reported in endocrine and neuronal cells exposed to hyperglycaemia [45, 46]. Moreover we found a lower expression of Syntaxin-1 and SNAP-25 in pancreatic beta-cells cultured with GS [47]. Consistently with these results, we found that the defective GLP-1 secretion in GLUTag cells exposed to GS may be due to the altered expression of these proteins. Since the intracellular protein content of SYNTHAXIN-1 was downregulated by both GS and HG and SNAP-25 was decreased only in cells cultured with GS, our results suggest that mainly SNAP-25 is critical for GLP-1 exocytosis.

Another main result of the present study is represented by the identification of GS and HG as regulators of glucose metabolism and insulin resistance in GLUTag cells. The different regulation of the enzyme glucokinase (increased only by HG) might suggest a differential regulation of the cell glucose metabolism. According to the observation of Reimann and Gribble [42] the increased expression of GK in GLUTag cells exposed to HG may improve the glycolytic flux and, consequently, ATP generation leading to upregulation of GLP-1 secretion. On the other hand, we found that high levels of GS significantly decreased insulin responsiveness in GLUTag cells and that this effect is independent of IRS-1 expression. Interestingly, these results suggested that GS might induce insulin resistance also in cells which are not considered classical targets for insulin-mediated activities. Furthermore, since insulin was already shown to regulate GLP-1 release from L cells [13, 48], our results suggest that the negative correlation between insulin resistance and GLP-1 secretion [9, 11] might also involve insulin resistance in cells secreting GLP-1.

## 5. Conclusions

In conclusion, this study demonstrated that function of GLP-1 secreting cells may be affected by the “diabetic milieu.” Although highly speculative due to the fact that these experiments are limited to *in vitro* cell cultures, stimulation with GS was associated with increased ROS production, upregulation of proglucagon mRNA levels, and reduction in proglucagon processing. On the other hand, HG was associated with increase in ROS production, proglucagon mRNA expression, GLP-1 production and secretion, and expression of PC 1/3 and GK. Furthermore, our results demonstrated that the “metabolic memory” may be more deleterious than hyperglycaemia per se, suggesting that even in presence of euglycaemic condition previous glycaemic uncontrolled excursions have to be carefully considered as detrimental in diabetes complications.
